# The Effect of Excess Electron and hole on CO_2_ Adsorption and Activation on Rutile (110) surface

**DOI:** 10.1038/srep23298

**Published:** 2016-03-17

**Authors:** Wen-Jin Yin, Bo Wen, Sateesh Bandaru, Matthias Krack, MW Lau, Li-Min Liu

**Affiliations:** 1 Beijing Computational Science Research Center, Beijing 100084, China; 2 Paul Scherrer Institute, CH-5232 Villigen-PSI, Switzerland; 3 Chengdu Green Energy and Green Manufacturing Technology R&D Center, Chengdu, Sichuan, 610207, China

## Abstract

CO_2_ capture and conversion into useful chemical fuel attracts great attention from many different fields. In the reduction process, excess electron is of key importance as it participates in the reaction, thus it is essential to know whether the excess electrons or holes affect the CO_2_ conversion. Here, the first-principles calculations were carried out to explore the role of excess electron on adsorption and activation of CO_2_ on rutile (110) surface. The calculated results demonstrate that CO_2_ can be activated as CO_2_ anions or CO_2_ cation when the system contains excess electrons and holes. The electronic structure of the activated CO_2_ is greatly changed, and the lowest unoccupied molecular orbital of CO_2_ can be even lower than the conduction band minimum of TiO_2_, which greatly facilities the CO_2_ reduction. Meanwhile, the dissociation process of CO_2_ undergoes an activated CO_2_^−^ anion in bend configuration rather than the linear, while the long crossing distance of proton transfer greatly hinders the photocatalytic reduction of CO_2_ on the rutile (110) surface. These results show the importance of the excess electrons on the CO_2_ reduction process.

The increasing industrial growth has led to accelerated energy consumption especially the traditional fossil fuel, which inevitable releases amount of CO_2_ that results in seriously global warming problem. It is of great urgency to reduce CO_2_ emission, and this problem is gaining plenty of attentions from various fields^1^,^2^,^3^. In addition to the biological photosynthesis, different strategies such as physical and chemical approaches have been proposed to reduce and convert CO_2_ to chemical fuels^4^,^5^,^6^. Photo-catalytic CO_2_ conversion has been proved to be an efficient way to convert CO_2_ by harnessing renewable solar energy, and it will generate synthetic fuels such as formaldehyde (HCHO), formic acid (HCOOH), methanol (CH_3_OH), and methane (CH_4_)^7^,^8^,^9^.

Titanium dioxide (TiO_2_) is considered as a model photocatalysis for CO_2_ conversion as it is highly stable, nontoxic and cheap^10^,^11^. The early experiment proposed by Inoue *et al.*, reported that under UV light photo-catalytic reduction of CO_2_ in the aqueous suspension of photosensitive semiconductor powders can form HCHO, HCOOH, CH_3_OH, and CH_4_ as main products^7^. Later, many efforts have been devoted to increase the efficiency and selectivity of the photo-catalytic CO_2_ reduction^12^,^13^,^14^,^15^,^16^. The photo-catalytic CO_2_ reduction results suggest that the catalytic activity can also be affected by TiO_2_ phase, and they found that the brookite has a much higher activity than the anatase or rutile phase^17^,^18^. Further, the efficiency and selectivity of CO_2_ reduction can be improved through doping noble metals, such as Pt, Pd, Cu, and Au atoms on TiO_2_^19^,^20^,^21^,^22^. A previous report showed that the efficiency of CO_2_ conversion into fuels can also be significantly affected by the hole-sacrifice, such as methanol^23^. Nonetheless, CO_2_ can be successfully converted through photo-catalytic reduction, both the efficiency and selectivity of photo-catalytic system are still too low and poor for the realistic application. In order to design a more efficient and selective photo-catalyst, it is important to understand the detailed CO_2_ reduction mechanism at the molecular level.

The photo-catalytic reduction of CO_2_ into synthetic fuels is a multiple electron reaction process, which involves two-electron process to form CO and HCOOH, four-electron process to form HCHO, and eight-electron process to form CH_4_^3^,^24^,^25^,^26^. For all these multiple electron reaction processes, the process starts initially from the adsorption and activation of CO_2_ molecule, in which CO_2_ involves configuration transformation, such as linear CO_2_ to bent and proton transfer takes place. In real reaction process, the activation of CO_2_ is a rate-limiting step^5^. CO_2_ is a rather inert molecule with a positive electron affinity of 0.6±0.2 eV^27^. Meanwhile, with respect to the normal hydrogen electrode (NHE), the reduction potential of CO_2_/CO_2_^−^ is about 1.9 eV, which is much higher than the TiO_2_ conduction band minimum (CBM) about 0.4 eV above the Fermi level^28^. Thus, such relatively high potential prevents the efficient electron transfer process from TiO_2_ to CO_2_, which is a necessary for the photo-catalytic reduction reaction.

On the other side, several experiments have shown that the CO_2_ can be triggered on the pure TiO_2_^6^,^29^. The vibrational spectroscopic techniques have shown that CO_2_^−^ anions is identified on pure TiO_2_ surface, indicating electron can be transferred from TiO_2_ to CO_2_^23^. Additionally, Tan *et al.* found that CO_2_ molecule can be activated by one electron and reduced to CO on the reduced rutile (110) surface based on scanning tunneling microscopy^30^. How to reconcile this paradox as most of the experimental results appear CO_2_ can be converted, while lowest unoccupied molecular orbital (LUMO) value of CO_2_ molecule is extremely high^28^. On the theoretical side, He *et al.* reported the CO_2_^−^ anion is one of the important species on the charged anatase (101) surface, and the reduction of CO_2_ into HCOOH or CO mainly it takes 2e^−^ reaction on anatase TiO_2_(101)^31^,^32^. Thus, it is urgency to know how the excess electrons effect on the CO_2_ adsorption and activation during the reduction process at the molecular level.

In this paper, we explore excess electrons effect on the structure and reactivity of CO_2_ on the perfect and reduced rutile (110) by first-principles calculations. Spin moment and density calculations show that the CO_2_ anion can exist in the TiO_2_ (110) containing excess electrons, and a new configuration of CO_2_ cation exists in the hole system. Furthermore, the electronic density of various CO_2_ adsorptions show that the LUMO of CO_2_ can be tuned by the excess electrons or hole. Especially, the LUMO of the activated CO_2_ can even be lower than the TiO_2_ CBM, which can effectively lower the reaction barrier. Our results show that the CO_2_ activation and reduction processes on the rutile (110) surface are greatly affected by the excess electrons and holes.

## Results

In the present study, we examine the effect of excess electrons on the CO_2_ adsorption and activation on the perfect/reduced rutile (110) surfaces. We firstly focus on the role of excess electrons on the CO_2_ adsorption configurations adsorbed on perfect rutile (110) surface. Later, intrinsic oxygen vacancy (O_v_) defect is further explored. Further, we explore reaction pathway of CO_2_ dissociation into CO on O_v_ rutile (110) surface and mechanism involves photo-catalytic reduction of CO_2_ to form a HCOOH.

### The excess electrons effect on CO_2_ adsorption on the perfect rutile (110) surface

**Figure 1:**
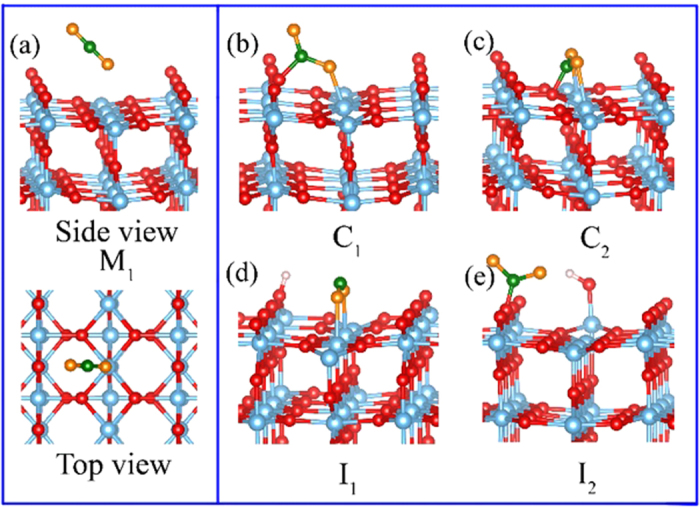
Different adsorption configurations of CO_2_ adsorbed on excess electrons perfect rutile (110) surface. (**a**) M_1_, molecular CO_2_ adsorbs at five-fold Ti^5f^ in a tilted style. (**b**) C_1_, one O atom of CO_2_ bonds sits at the fivefold Ti^5f^, and the C atom of CO_2_ bonds with the bridge oxygen. (**c**) C_2_, the two O atoms of CO_2_ adsorb at two adjacent Ti^5f^ and the C atom bonding with the O^3f^ atom of TiO_2_. (**d**) I_1_, quite similar to C_2_, while the C atoms does not interact with the surface oxygen atoms. (**e**) I_2_, the C atom of CO_2_ adsorbs on the top of the bridging oxygen in a tilted style. The O and Ti atoms in TiO_2_ are represented in red and gray blue balls, while O and C atoms in CO_2_ molecule are represented in orange and green balls.

In this section, we initially focus on the possible CO_2_ adsorption configurations in the case of excess electrons on the perfect rutile (110) surface. Before discussing the detailed CO_2_ adsorption, it should be emphasized that the linear molecular CO_2_ is firstly physically adsorbed on the rutile (110) surface. And according to the previous our results^33^, the molecular CO_2_ linearly adsorbed at five-fold Ti^5f^ in a tilted style is the most stable one. Based on this adsorption, the molecular CO_2_ will undergo a translation into bend through activation or reduction. As a result, the molecular adsorbed CO_2_ changes to the bend chemical adsorption. Five different binding configurations of CO_2_ exist on rutile (110) surface. All possible adsorption configurations are examined, which are labeled as M_1_, C_1_, C_2_, I_1_ and I_2_ (see Fig. 1). M_1_ is a physical adsorption, where the CO_2_ linearly adsorbs at five-fold Ti^5f^ in a tilted style. Except M_1_, all other four C_1_, C_2_ and I_1_, I_2_ configurations are chemical adsorptions: In C_1_ configuration, one O atom of CO_2_ bonds to the fivefold Ti^5f^, and the C atom of CO_2_ interacts with the bridge oxygen, forming a bent CO_2_ configuration; As for C_2_, the two O atoms of CO_2_ adsorbs at two adjacent Ti^5f^ sites, and the C atom directly bonds with the O^3f^ atom of TiO_2_; Quite similar to C_2_, the C atom of I_1_ does not interact with surface oxygen atom; As for I_2_, the CO_2_ adsorbs on the top of the bridging oxygen, forming a new C-O^2f^ bond.

**Figure 2:**
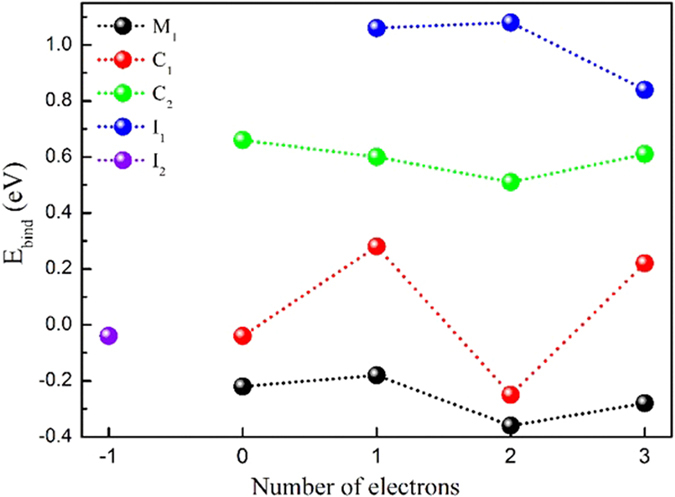
The binding energy of the CO_2_ adsorptions vs the different number of excess electrons in prefect rutile (110) surface. The positive digit denotes the number of excess electrons, and the negative digit means the number of hole in the transverse axis. Here the negative value indicates relatively large adsorption energy.

In Fig. 2, the positive digit in the transverse axis represents the number of excess electron, while the negative digit denotes the number of hole. When the system does not contain any excess electron or hole, three different binding configurations of CO_2_ on the perfect TiO_2_ were identified after the geometry relaxation, namely M_1_, C_1_ and C_2_. The others, I_1_ and I_2_, are unstable. The corresponding binding energy shows that M_1_ has the largest binding energy of −0.23 eV as the system does not contain excess electron, which is a little smaller than the earlier reported pure PBE value of −0.35 eV^33^,^34^. This is because PBE+U functional treats the d-orbital of Ti in a more localization.

As an electron is introduced to TiO_2_, I_1_ can exist with a binding energy of 1.02 eV. Therefore, the CO_2_ adsorption in I_1_ is meta-stable. It should be noted that the same kind configuration is also reported on the anatase (101) surface, and the corresponding adsorption energy of CO_2_ is 0.78 eV^31^, which is quite close the current one. When more electrons are included in TiO_2_, no new configuration appears, and the corresponding binding energy are also not sensitive to the number of excess electrons. When the TiO_2_ surface is charged with holes, the configuration of I_2_ can exist with a binding energy of −0.05 eV.

The detailed geometrical parameters, net charge, and spin polarized moment of the CO_2_ adsorption configurations are also calculated as shown in Table 1. Among these five states, the configurations of M_1_, C_1_ and C_2_ adsorbed on the perfect without excess electron or hole are chosen as they are not sensitive to the excess electrons. While the configurations of I_1_ and I_2_ are shown for the system containing one electron or hole. Compared with the single CO_2_ molecule, the C-O bond length of CO_2_ in M_1_ is almost similar, and the ∠O-C-O angle slightly decreases by 2.38°. The net charge and spin polarized moment of adsorbed CO_2_ are the same to that of single CO_2_ molecule. A keen look into the structures of the C_1_/C_2_, the C-O bond length in C_1_/C_2_ increases by 0.08/0.14 Å, while the ∠O-C-O bond angle decreases by 53.76/47.45°. In case of C_1_ and C_2,_ owing to the strong interaction between the CO_2_ and rutile (110) surface, charge transfer occurs from TiO_2_ to CO_2_ by about 0.29e^−^. The spin polarized moment of these two adsorbed CO_2_ states is zero, demonstrating that there is no unpaired electron existing in both C_1_ and C_2_.

**Figure 3:**
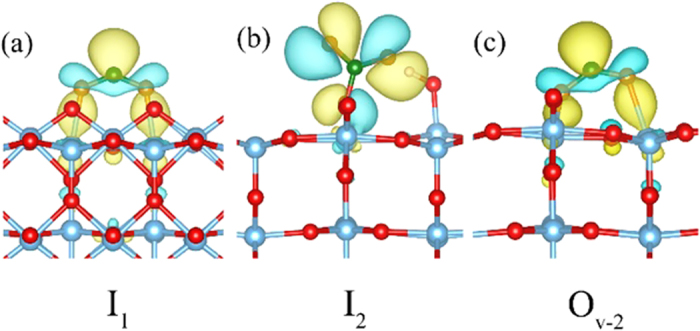
Spin densities of different binding configurations of CO_2_ on excess electrons perfect and O_v_ rutile (110) surface. Spin density of (**a**) I_1_ with an excess electron and (**b**) I_2_ with an hole on the perfect rutile (110) surface. (**c**) spin density of O_v-2_ on the reduced rutile (110) surface.

As we mentioned above, when the system contains the excess electron, the I_1_ becomes metastable. As for the I_1_, the relaxed geometric parameters (Ti-O, C-O and ∠O-C-O) of I_1_ and net charges are quite close to C_2_, but the spin polarized moment is 0.74 μ_B_, indicating an unpaired electron is located on the CO_2_ forming a activated state of I_1_. The corresponding spin densities as shown in Fig. 3. The excess electron is mainly localized on the C atom of the CO_2_, suggesting that an excess electron is transferred from TiO_2_ to CO_2_ and to form a CO_2_^−^ anion^31^. It should be mentioned that although this configuration is rather unfavorable, the extra electron is shown to be critical to stabilize this binding configuration. When it comes to I_2_, the C-O bond length is little elongated to 1.27 Å, and the ∠O-C-O angle enormously decreases to 120.27°. The spin polarized moment is about 0.90 μ_B_, indicating an unpaired electron is located on CO_2_. Further spin density calculation demonstrates that the electron in I_2_ is localized at the two O atoms instead of C atom in the CO_2_ forming a activated CO_2_^+^ cation as shown in Fig. 3, which is different from the previous reported result only forming CO_2_^−^ state^31^. From the above results, we clearly observe that various CO_2_ adsorptions appear on the perfect rutile (110) surface in the case of excess electrons or holes.

### CO_2_ adsorption on O_v_ rutile (110) surface

Apart from excess electron on the perfect TiO_2_ case, the intrinsic oxygen vacancy (O_v_) defect can also provide two excess electrons to the rutile TiO_2_(110)^35^. Here, we consider one O_v_ defect in rutile (110) surface to simulate the effect of excess electrons on the CO_2_ adsorption. Relative to the above perfect TiO_2_, O_v_ defect not only provides the excess electrons but also the adsorption sites.

**Figure 4:**
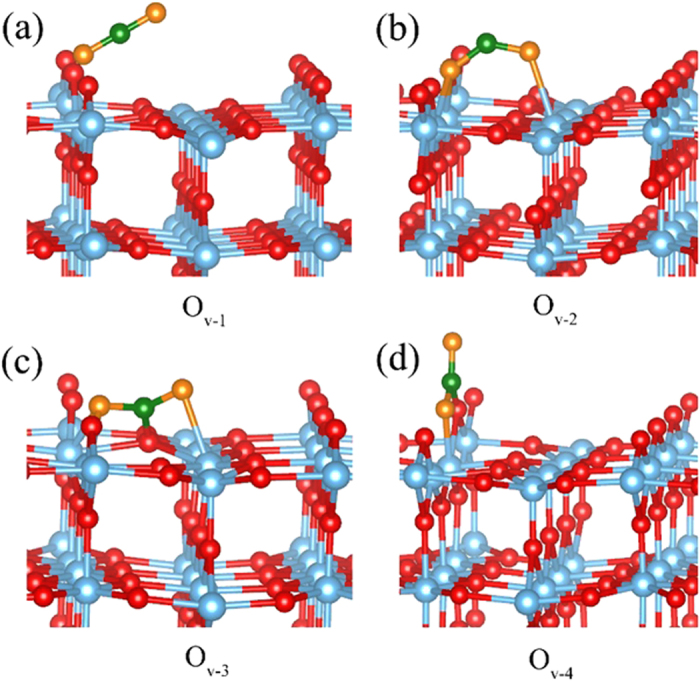
Different adsorption configurations of CO_2_ on O_v_ rutile (110) surface. (**a**) O_v-1_, CO_2_ molecule adsorbed at O_v_ site in a titled style. (**b**) O_v-2_, one O of CO_2_ adsorbed at O_v_ site and the other O adsorbed at Ti^5f^ in the plane. (**c**) O_v-3_, quite similar to O_v-2_ except for the C atom linked to O^3f^ in the plane. (**d**) O_v-4_, one O atom of CO_2_ adsorbed at O_v_ site and the C atom adsorbed with the O^2f^ near the O_v_ site.

Here, four different configurations are examined, labeled as O_v-1_ ∼ O_v-4_ as shown in Fig. 4. O_v-1_ linearly adsorbs in the middle of O_v_ in a tilted configuration. In case of O_v-2_, one of the O atom of CO_2_ binds to two 5-fold Ti atoms (Ti^5f^) in the O_v_ site through bi-dentate fashion, while the other “O” atom interacts with the 5-fold Ti atom (Ti^5f^) in the plane. The configuration of O_v-3_ is quite close to O_v-2_ except for the C atom linked to the 3-fold “O” atom (O^3f^) in the plane; the CO_2_ in O_v-4_ adsorbs in the bridging oxygen row with the O mono-dentate adsorbed to Ti^5f^ in O_v_ site and C atom bonds to bridging oxygen O^2f^.

The corresponding binding energies, geometrical parameters, net charge, and spin polarized moment of the CO_2_ adsorption on reduced TiO_2_ are also summarized in Table 1. Previous theoretical result shows that the CO_2_ interacting with O_v-4_ configuration on the anatase (101) cluster is the most stable adsorption with the binding energy of −1.09 eV^31^. Similarly, the calculated binding energy of CO_2_ in O_v-4_ has the highest binding energy of −1.11 eV in our present study, indicates that O_v-4_ is indeed more favorable adsorption. The binding energy of O_v-1_ is about −1.08 eV, which is quite close to O_v-4_, suggesting that O_v-1_ is also relatively stable configuration. The other two configurations O_v-2_ and O_v-3_ have relatively lower binding energies than O_v-1_ and O_v-4_, indicating that they are meta-stable.

A keen look into the geometrical parameters of O_v-1_~O_v-4_, the CO_2_ in O_v-1_ configuration both bond lengths and angles are very close to the isolated CO_2_ molecule (Table 1). Unlike the O_v-1_, the bond length of C-O in O_v-2_~O_v-4_ is elongated by 0.1–0.16 Å, and the angle ∠O-C-O significantly decreases by ∠47.05–50.68° compared with an isolated CO_2_ molecule. Similar to C_1_ and C_2_ on perfect TiO_2_ (110), net charge of O_v-2_~O_v-4_ is about −0.41e^−^, suggesting that charge redistribution between CO_2_ and TiO_2_. Most strikingly, spin polarized moment studies shows that the O_v-1_, O_v-3_, and O_v-4_ the calculated spin moments are equal to zero, whereas O_v-2_ has a spin moment of 0.82 μ_B_, indicating an electron is located in the CO_2_. Further spin density calculation reveals that the electron is localized on the C atom of the CO_2_ as shown in Fig. 3. Thus, the CO_2_ in O_v-2_ indeed converts into an activated CO_2_^−^ anion. Although O_v-2_ has a relatively lower binding energy than other configurations, the extra electron at “C” atom is crucial to stabilize the binding configuration.

**Figure 5:**
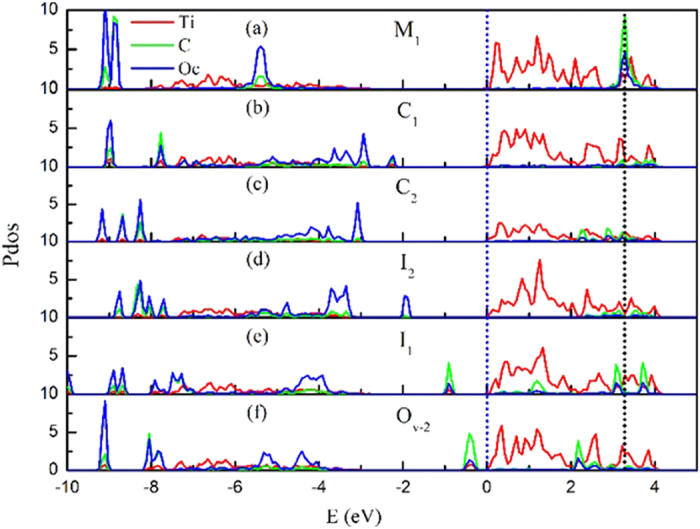
The partial density of states (PDOS) of the different CO_2_ adsorption configurations on the excess electrons rutile (110) surface. (**a**) PDOS for the molecular CO_2_ adsorption state M_1_, and (**b**)/(**c**) corresponds to bending adsorption state C_1_/C_2_. (**d**,**e**) PDOS for I_2_ with an hole and I_1_ with an excess electron, respectively. (**f**) PDOS for O_v-2_ adsorbed on reduced rutile (110). The plots in red is the PDOS of the single Ti atom adsorbed by the CO_2_. The plots in green and blue are the C atom and O atom of the adsorbed CO_2_, respectively.

It is well known that the LUMO value of an isolated CO_2_ molecule is very high, and the electron is very difficult to transfer to the CO_2_ molecule from the TiO_2_ conduction band^3^. In order to know whether the above CO_2_ adsorptions can affect the LUMO of CO_2_ in the presence of excess electron or hole, the partial density of states (PDOS) of the adsorbed CO_2_ is calculated. The results are shown in Fig. 5. As for M_1_, the LUMO value is located above the TiO_2_ CBM onset by 3.4 eV. This value is in consistent with the estimated value of 3.5 eV by Indrakanti *et al.*, and a little larger than the value of 2.3 eV by Tan *et al.*^3^,^30^. Thus, the electron in the TiO_2_ CBM is rather difficult to be transferred to the CO_2_ in molecular state. When the CO_2_ is changed to bending adsorption configurations (C_1_ and C_2_), the localized LUMO of CO_2_ molecule becomes delocalized state, and the LUMO onset shifts down to 2.3 eV. Therefore, the energy level can be modified by CO_2_ adsorption mode. Whereas this value is still too large for the electron transfer from the TiO_2_ conduction band to the CO_2_ molecule. When the CO_2_ adsorptions with the configurations of I_1_, I_2_, and O_v-2_ on TiO_2_ (110) containing excess electron or hole, the PDOS shows the LUMO of CO_2_ shifts further downward, which can even be lower than the TiO_2_ CBM. Hence, the electron or hole can easily transfer from TiO_2_ CBM to the CO_2_ with I_1_, I_2_, and O_v-2_.

### Dissociation of CO_2_ into CO on O_v_ rutile (110) surface

As discussed above, the CO_2_ adsorption on the O_v_ rutile (110) surface can be activated, and the corresponding LUMO is even lower than TiO_2_ CBM. Thus it is interesting to know how the CO_2_ adsorption on O_v_ rutile (110) can be further converted into the other species. The activation process can be expressed as:


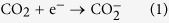


**Figure 6:**
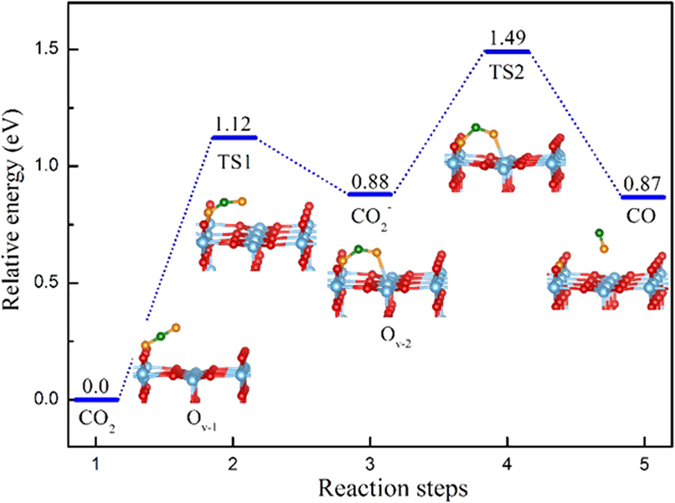
Illustration of reaction pathway via O_v-2_ configuration to form CO. The sum energy of the CO_2_ and O_v_ rutile TiO_2_ is the zero reference for energy.

From the equation (2), we can clearly observe that the activation of CO_2_ process needs an excess electron in the system. Scanning tunneling microscopy experiment suggested that the conversion of CO_2_ to CO is relative to electron attachment state of linear CO_2_ molecule on the O_v_ rutile (110) surface^30^. However, the detailed dissociation of CO_2_ mechanism at molecular level is still unknown. Here, we start the study from configuration O_v-1_ with intrinsic excess electrons in reduced TiO_2_, and the dissociation of CO_2_ into CO are explored. The detailed reaction pathway and calculated energy barriers are shown in Fig. 6.

As shown in Fig. 6, the CO_2_ molecule firstly adsorbs at the oxygen vacancy, forming the linear adsorption as O_v-1_. In this step, there is no electron transfer from reduced TiO_2_ to linear CO_2_, which is a different from the previous result where the linear CO_2_ can form an electron attachment state^36^. Then, the linear adsorbed CO_2_ molecule initiates to bent, which undergoes a transition state transition state 1 (TS1) with an energy barrier 1.12 eV to form O_v-2_ structure. Subsequently, the excess electron in the TiO_2_ transfers to the CO_2_, forming CO_2_^−^ anion (see Fig. 3). On the basis of CO_2_^−^ anion, the C-O bond breaks to form CO, leaving an O atom at the oxygen vacancy site. The energy barrier of this process is about 0.61 eV. From the whole process, the CO formation undergoes an activation state of O_v-2_ rather than a direct C-O bond breaking of linear CO_2_.

### Photo-catalytic reduction of CO_2_ to form HCOOH

Apart from the CO formation in the reduced TiO_2_, photo-catalytic reduction of CO_2_ can also form synthetic fuels such as formaldehyde (HCHO), formic acid (HCOOH), methanol (CH_3_OH), and methane (CH_4_) on the TiO_2_ based materials. However, the formation of these useful fuels through activated CO_2_ is still rare, and most of the theoretical researches mainly focus on the anatase phase^32^. Here, on the basis of configuration I_1_, the reduction of CO_2_ to form HCOOH is investigated, which can be expressed as:


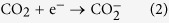



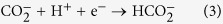



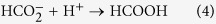


From equations (3) to (5), we can clearly understand that the reaction involves two electrons and two protons in the system. The complete reduction process can be divided into five steps as follows: The molecular CO_2_ firstly adsorbs on the rutile (110) surface as C_1_; Following this, excess electron injects into CO_2_, and the corresponding CO_2_ becomes CO_2_^−^ anion as I_1_; Then, one proton and electron transfer to the CO_2_^−^ anion, forming HCO_2_^−^; Finally, the other proton transfers to HCO_2_^−^, forming HCOOH.

**Figure 7:**
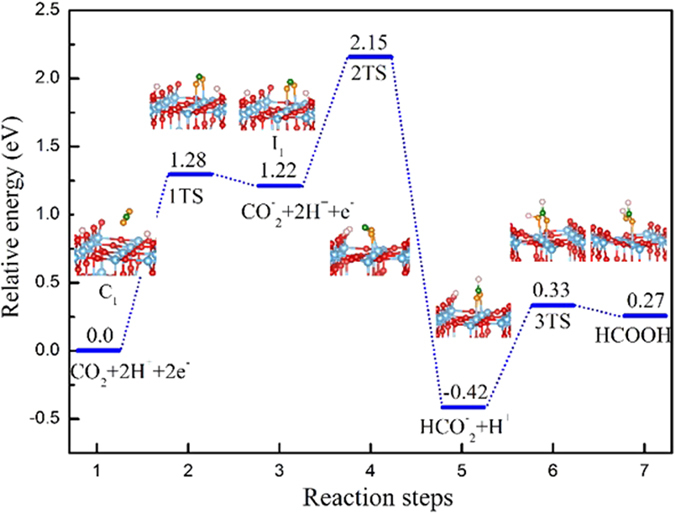
Illustration of reaction pathway via I_1_ configuration to form HCOOH. The sum of energies of the CO_2_ and 2H is the zero reference for energy. The sign of “ + ” indicates non-interacting species (e.g. CO_2_ + OH), and the transition state denotes by TS.

According to the above reaction processes, the transition states and corresponding energy barriers of CO_2_ reduction to HCOOH are summarized in Fig. 7. On the basis of configuration C_1_, the two O atoms of CO_2_ begin to bend towards the adjacent fivefold Ti^5f^ in plane through TS1, the two O atoms of CO_2_ is adsorbed by Ti^5f^ in nature as shown step-3 in Fig. 7. Consecutively, the electron spontaneously transfers to the CO_2_ forming the CO_2_^−^ anion. This process needs to overcome an energy barrier of 1.28 eV, which is much higher than the case in anatase (101) of 0.87 eV^32^. Consecutive proton and electron move to the “C” atom of CO_2_^−^ anion to form HCO_2_^−^. These findings are very different from the case in anatase (101). In anatase case, proton transfer occurs with no energy barrier, but the proton transfer on rutile needs to overcome an energy barrier of 0.93 eV. The calculated energy barrier is relatively higher, because the proton should move about 3.10 Å between the adsorbed proton and the CO_2_^−^ anion, which is larger than the one in anatase (about 2.60 Å). Further, the other proton moves to the “O” atom of HCO_2_^−^, resulting in HCOOH. This process needs a moderate energy barrier of 0.75 eV. From the complete reduction process, the formation of CO_2_^−^ anion is the rate limiting step, and also the proton transfer step is much difficult than the earlier reported anatase (101) surface^32^.

### Activation of CO_2_ by a hole

On the basis of configuration I_2_, the activation of CO_2_ by the hole is also investigated, which can be expressed as:


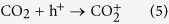


**Figure 8:**
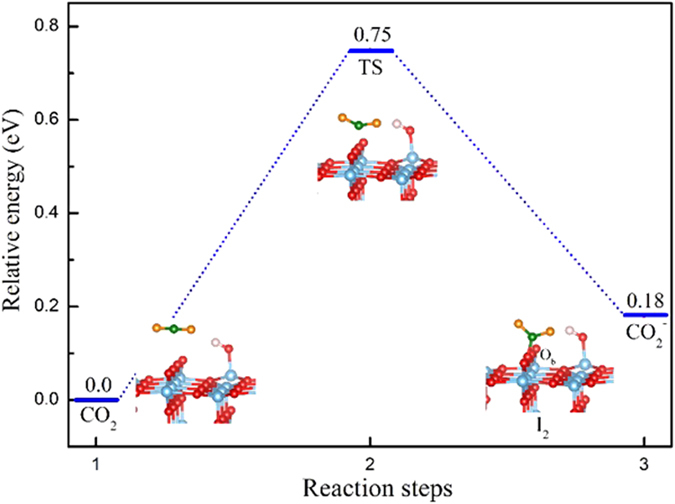
Illustration of reaction pathway to form CO_2_^+^ cation. The sum energy of CO_2_ and TiO_2_ is set to zero as the reference energy.

In the CO_2_ activation process (Eq. 6), a hole is transferred to the O atom of CO_2_ in configuration I_2_. The detailed reaction process and activation barrier of hole to CO_2_ with I_2_ are calculated (Fig. 8). The molecular CO_2_ adsorbs on the top of bridge oxygen on TiO_2_ with a C-O_b_ distance of 2.71 Å. Then the C atom of the CO_2_ moves towards the bridge-site oxygen. In the transition state geometry, the C-O_b_ distance decreases from 2.71 Å to 1.74 Å. Finally, the “C” atom of CO_2_ adsorbed with the bridge oxygen forming a bent like CO_2_, and the C-O_b_ distance decreases to 1.34 Å. Simultaneously, the hole is transferred to the two “O” atoms of CO_2_ forming a CO_2_^+^ as shown in Fig. 3. Formation of cation need to overcome a relatively lower energy barrier (about 0.75 eV) than that of anions.

## Discussions

In summary, by the first-principles calculations, structural and reactivity behavior of CO_2_ on rutile TiO_2_ are greatly affected by excess electrons. The computed results show that various CO_2_ adsorption configurations appear in the case of excess electrons, and activated CO_2_ adsorption configurations can be exist in the not only excess electron system as I_1_ and O_v-2_, but also in the hole system as I_2_. Further electronic density calculation shows that the LUMO of CO_2_ can be modified by varying the CO_2_ adsorption states, and it can even be lowered and below the TiO_2_ conduction band. The detailed CO_2_ activation and reduction processes are also explored. The mechanism of CO_2_ reduction to CO on oxygen vacancy rutile (110) surface is revised, the reduction process involves the formation of CO_2_ anion in bend type structure with an energy barrier of 1.12 eV. The results also suggest that, the energy barrier of rate limitation step to form HCOOH is about 1.28 eV. In addition, the process for the formation of CO_2_^+^ cation in the hole system is also investigated, and it needs a much lower energy barrier of 0.75 eV.

## Method

The calculations are performed based on the spin-polarized density functional theory (DFT) in periodic boundary conditions, as implemented in the CP2K/Quickstep package^37^. This simulation code employs hybrid Gaussian and plane wave (GPW) basis sets and norm conserving Goedecker-Teter-Hutter (GTH) pseudo-potentials to represent the ion-electron interactions^38^,^39^. The Gaussian functions consisting of a double-ζ plus polarization (DZVP) basis set was employed to optimize the structures^40^. The energy cutoff for the real space grid was 500 Ry, which yields total energies converged to at least 0.001 eV per atom. For the exchange-correlation functional, we have used the Perdew-Burke-Ernzerhof (PBE) functional of generalized gradient approximation (GGA)^41^. The vdW correction is considered with the Grimme approach (DFT-D3)^42^. Since the standard GGA functional has the limitation to calculate the d-band electrons of transition metal, GGA+U functional is used to treat Ti 3d electron with U = 4.2 eV^43^. In order to avoid the interaction between the adjacent images, a vacuum spacing of 15 Å is employed for all the systems. Transition states along the reaction pathways are searched by the Climbing Image Nudged Elastic Band (CI-NEB) approach^44^.

The interaction between the adsorbed molecule and the substrate, which can be characterized by the binding energy, which is defined as,


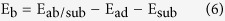


where E_ad/sub_ is the total energy of the molecule adsorbed on the substrate, E_ad_ is the energy of the isolated molecule in the same box, and the E_sub_ is the energy of the substrate. In the present study, a (4 × 2) supercell is used to represent rutile TiO_2_ (110) substrate containing four tri-layers. In the rutile TiO_2_(110) features three types of under coordinated atoms: five-fold Ti ionic (Ti^5f^), bridge oxygen atom in two-fold (O^2f^), and planar three-fold oxygen atom (O^3f^). The excess electrons in the system are simulated by adding hydrogen atoms or hydroxyls, and one hydrogen/hydroxyl corresponds to one electron/hole^45^. All the CO_2_ adsorption configurations studied in the text, only one CO_2_ molecule is considered to adsorb on the (4 × 2) supercell, corresponding to 1/8 ML coverage.

## Additional Information

**How to cite this article**: Yin, W.-J. *et al.* The Effect of Excess Electron and hole on CO_2_ Adsorption and Activation on Rutile (110) surface. *Sci. Rep.* **6**, 23298; doi: 10.1038/srep23298 (2016).

## Figures and Tables

**Table 1:** Representative geometrical parameters, net charge, spin polarized moment for the CO_2_ adsorbed on excess electron perfect (M_1_, C_1_, C_2_, I_1_, and I_2_) and reduce rutile (110) (O_v-1_, O_v-2_, O_v-3_, and O_v-4_) surface.

Parameter	M_1_	C_1_	C_2_	I_1_	I_2_
Ti-O (Å)	2.60	1.94	2.16	2.13	
C-O (Å)	1.16	1.32	1.26	1.25	1.26
O-C-O (θ)	177.6	127.2	132.5	135.0	120.2
Net Charge (e)	0.05	−0.29	−0.29	−0.29	−0.05
Spin (μ_B_)	0	0	0	0.74	0.90
**Parameter**	**CO**_**2**_	**Ov-1**	**Ov-2**	**Ov-3**	**Ov-4**
Ti-O (Å)		2.67	2.23	2.15	2.70
C-O (Å)	1.18	1.19	1.28	1.29	1.34
O-C-O (θ)	180.0	179.8	132.9	129.3	129.3
Net Charge (e)	0	0.06	−0.41	−0.43	−0.35
Spin (μ_B_)	0	0	0.82	0	0
E_bind_ (eV)		−1.08	−0.16	−0.83	−1.11

The binding energy for CO_2_ on reduced TiO_2_ is also included.
